# Iron Deficiency in Obesity and after Bariatric Surgery

**DOI:** 10.3390/biom11050613

**Published:** 2021-04-21

**Authors:** Geir Bjørklund, Massimiliano Peana, Lyudmila Pivina, Alexandru Dosa, Jan Aaseth, Yuliya Semenova, Salvatore Chirumbolo, Serenella Medici, Maryam Dadar, Daniel-Ovidiu Costea

**Affiliations:** 1 Council for Nutritional and Environmental Medicine, Toften 24, 8610 Mo i Rana, Norway; 2 Department of Chemistry and Pharmacy, University of Sassari, Via Vienna 2, 07100 Sassari, Italy; sere@uniss.it; 3 Department of Neurology, Ophthalmology and Otolaryngology, Semey Medical University, 071400 Semey, Kazakhstan; semskluda@rambler.ru (L.P.); yuliyasemenova@yahoo.com (Y.S.); 4 CONEM Kazakhstan Environmental Health and Safety Research Group, Semey Medical University, 071400 Semey, Kazakhstan; 5 Faculty of Medicine, Ovidius University of Constanta, 900470 Constanta, Romania; dosa.alexandru@gmail.com (A.D.); danielocostea@gmail.com (D.-O.C.); 6 Research Department, Innlandet Hospital Trust, 2380 Brumunddal, Norway; jaol-aas@online.no; 7 Department of Neurosciences, Biomedicine and Movement Sciences, University of Verona, 37134 Verona, Italy; salvatore.chirumbolo@univr.it; 8 CONEM Scientific Secretary, 37134 Verona, Italy; 9 Razi Vaccine and Serum Research Institute, Agricultural Research, Education and Extension Organization (AREEO), Karaj 31975/148, Iran; dadar.m77@gmail.com

**Keywords:** obesity, iron deficiency, iron metabolism, bariatric surgery, iron supplement

## Abstract

Iron deficiency (ID) is particularly frequent in obese patients due to increased circulating levels of acute-phase reactant hepcidin and adiposity-associated inflammation. Inflammation in obese subjects is closely related to ID. It induces reduced iron absorption correlated to the inhibition of duodenal ferroportin expression, parallel to the increased concentrations of hepcidin. Obese subjects often get decreased inflammatory response after bariatric surgery, accompanied by decreased serum hepcidin and therefore improved iron absorption. Bariatric surgery can induce the mitigation or resolution of obesity-associated complications, such as hypertension, insulin resistance, diabetes mellitus, and hyperlipidemia, adjusting many parameters in the metabolism. However, gastric bypass surgery and sleeve gastrectomy can induce malabsorption and may accentuate ID. The present review explores the burden and characteristics of ID and anemia in obese patients after bariatric surgery, accounting for gastric bypass technique (Roux-en-Y gastric bypass—RYGB) and sleeve gastrectomy (SG). After bariatric surgery, obese subjects’ iron status should be monitored, and they should be motivated to use adequate and recommended iron supplementation.

## 1. Introduction

Iron (Fe) is one of about 20 essential trace elements bearing crucial functions in the human organism and almost all living systems [1,2,3]. Fe is a redox-active element highly widespread in the majority of human tissues, particularly, as it is well known, in muscle cells (myoglobin) and erythrocytes (hemoglobin) [4]. The human genome codes about 500 iron-containing proteins. Iron proteins, as ferritin (Ft) and hemosiderin, work as Fe storage proteins, whereas transferrin (Tf) acts as a Fe transporter in plasma. Furthermore, Fe is involved in several enzymatic processes and physiological reactions. Therefore, Fe is a fundamental trace element with well-controlled homeostasis [3]. Both iron deficiency (ID) and overload (IO) are related to common human diseases with different clinical symptoms, including anemia and siderosis, obesity, and even neurodegenerative disorders [5,6,7,8]. ID is known as the most common nutritional disorder globally. Between four and five billion people might suffer from ID and, because an estimated two billion are anemic, several hundred million manifest ID anemia [9,10,11,12]. ID is also associated with decreased work capacity in adults and impaired mental development in children. The latent ID without anemia can be severe, and specific laboratory tests are required for its detection, management, and diagnosis [13]. Commonly, ID and anemia can be induced by many factors such as helminthic infections, nutritional deficiencies, and irregular menstrual cycles in women. However, an inflammatory component is a critical factor in the different stages of uptake, storage, and transportation of Fe in anemia, which also is frequently observed in many inflammatory disorders [14].

This problem has a multifactorial nature, and the upregulation of hepcidin plays a key role. Hepcidin (Hep) is an inducer of innate immunity and an important component of inflammatory anemia. Hep can control the amount of bioavailable iron in acute inflammation; this limits inflammation and reduces erythropoiesis [15]. Inflammatory anemia is characterized by the fact that, with sufficient iron in the macrophages, the return of iron to serum is blocked by Hep. Interleukin-6 (IL-6) as a pro-inflammatory cytokine promotes Hep overproduction by activating the Janus kinase signal transducer and transcriptional activator 3. This leads to a degradation of ferroportin in lysosomes, slowing down iron transport into serum, its accumulation in macrophages, and decreasing iron transfer from enterocytes [16]. The inflammatory component of obesity leading to excessive production of Hep (and lipocalin 2) is considered one of the potential mechanisms of hypoferremia in obesity. The overproduction of these proteins is associated with the sequestration of iron in the cells of the reticuloendothelial system. In this case, iron accumulates in adipose tissue, causing oxidative stress and endocrine dysfunction of adipose tissue and inflammation of the endoplasmic reticulum. Iron-mediated mechanisms of toxicity can contribute to obesity aggravation. Thus, it is possible to explain the mutual influence of impaired iron status and the pathogenesis of obesity [10].

Cytokines IL-6, IL-1β, and IL-22 have been connected with the elevated Hep expression secondary to inflammation. In contrast, tumor necrosis factor α (TNFα), a key mediator of different inflammatory disorders, including inflammatory bowel disease (IBD), can inhibit Hep expression, and treatment with anti-TNFα antibodies improved anemia status in patients with IBD. Other “signals” that regulate Hep expression in the context of inflammation include endoplasmic reticulum stress and gastrointestinal microbiota composition [17].

Apart from Hep, inflammatory mediators can also influence iron homeostasis, and the sample of such mediators could be made of TNFα, which can act directly on intestinal epithelial cells to inhibit iron transport. Besides, a combination of lipopolysaccharide and pro-inflammatory cytokine interferon γ can result in intracellular sequestration of iron and decreased ferroportin levels [18].

Low-grade chronic inflammation is common in obesity, and recent research offered some insights into the intracellular pathways of obesity-associated inflammation. Such overfeeding is the starting point of inflammation, which originates from cells and tissues involved in metabolism, i.e., adipocytes and hepatic macrophages that trigger the inflammatory response. Liver tissues of obese individuals are characterized by increased activation of certain kinases (c-jun N-terminal kinase), with simultaneous inhibition of other kinases (k kinase) that can stimulate the expression of inflammatory cytokines. The upregulation of inflammatory mediator gene expression occurs due to downstream transcriptional programs, and the transcription factors involved are nuclear factor κB, activator protein-1, and interferon regulatory factor. Inhibitory signaling of metabolic pathways develops secondarily to increasing cytokines’ levels and exacerbating receptor activation [19]. The puzzling issue of ID associated with obesity is still under the spotlight, and the physiopathology of ID during obesity is not well defined. A relationship between obesity and the prevalence of ID has been recently investigated in adolescent people, showing that normal-weight individuals did not differ greatly from obese subjects (29.5% vs. 22.6%, *p* = 0.3) [20].

Consistent with several findings, obesity is significantly associated with ID [21,22,23,24,25], and according to some evidence, diet-induced weight loss could improve iron homeostasis and help obese people correct and resolve ID [26]. ID and its related symptoms (including pallor, fatigue, dry skin, brittle hair/nails, and loss of appetite) can occur not only if the Fe level is insufficient but also from its limited intestinal absorption or prolonged blood loss. Bariatric surgery in obese persons and individuals with Type 2 Diabetes (T2D) may cause ID [27].

ID in the human body after bariatric surgery occurs due to the following mechanisms:

- decrease in iron intake due to its low absorption
- poor tolerance to iron reach foods
- low adherence of patients to iron-containing drugs
- decreased hydrochloric acid secretion due to the presence of a shunt or resection of the greater curvature of the stomach
- decrease in the absorbing surface due to a duodenal shunt or damage to the intestinal villi.

Depending on the volume of surgery, further monitoring, dietary characteristics, in the long-term postoperative period, the prevalence of iron deficiency is detected in 18.0–53.3% of patients, and iron deficiency anemia reaches 52–54% [28]. Gastric bypass (Roux-en-Y gastric bypass—RYGB), sleeve gastrectomy (SG), adjustable gastric band (LAGB), and biliopancreatic diversion with duodenal switch (BPD/DS) are the most common surgical procedures used to cause weight loss in severely obese subjects and TD2 individuals. RYGB and SG are surgical procedures that induce a more rapid and significant long-term weight loss but could lead to vitamin and mineral deficiencies. ID and anemia resulting from Fe starvation have been reported in the range of 6–22% in obese patients referred to bariatric surgery, representing an elevated prevalence compared to 6–7% in the general population [29,30,31]. Furthermore, the anemia prevalence increases significantly after bariatric surgery, to about 10–63%, mostly attributed to iron deficiency [31,32,33,34]. However, iron deficiency is not synonymous with anemia, as vitamin B_12_ and B_9_ deficiencies can also provoke anemia. Apart from nutrient deficiencies, there are other potential causes of anemia, including inherited blood disorders, imbalances of certain hormones, hemolysis, and blood loss. The present review explores the burden and characteristics of ID and iron-deficiency anemia in obese patients and after bariatric surgery (especially RYGB and SG).

## 2. Iron Metabolism

Before addressing the role of obesity and bariatric surgery in causing or promoting ID, a brief introduction of iron physiology mechanisms is helpful. In addition to its well-known role in hemoglobin biosynthesis, Fe displays its function as a component of various proteins, explaining the variety of symptoms characterizing ID. Thus, Fe has an essential role in several metabolic, immunological, and central nervous functions [1,4,35,36,37].

Fatigue, weakness, depressed mood, and retardation of cognitive development in children can result from the altered metabolism of Fe [38,39,40,41]. The crucial proteins related to Fe metabolism are Hep, transferrin (Tf), and ferroportin-1 (Fpn-1) [42]. Genetic variations of these proteins have been closely associated with impaired iron metabolism, chronic anemia, and motor neuron disorder [41].

Hep and Fpn are two molecules mainly responsible for the regulation of systemic iron homeostasis. Balancing each other, they control the cellular iron export. Hep is an antimicrobial peptide of 25 amino acids, formerly known as LEAP-1 (liver-expressed antimicrobial peptide) [43]. Fpn, which exhibits 8-12 putative transmembrane domains, acts as a cellular iron exporter, while Hep reduces iron export by binding to Fpn, causing its subsequent degradation [44]. Hep inhibits the intestinal absorption and transfer of Fe from the duodenal enterocytes into blood plasma by inducing a change in the Hep receptor Fpn-1. The Hep-Fpn-1 complex is located in cells that have an essential role in degrading and internalizing the Fpn-1, and thus, the complex represses the Fe efflux from enterocytes, macrophages, and hepatocytes, reducing the Fe released into the circulation [42]. Thus, Hep is an inhibitor of the Fe uptake from the gut and the recycling of Fe from the reticuloendothelial system (RES).

**Figure 1:**
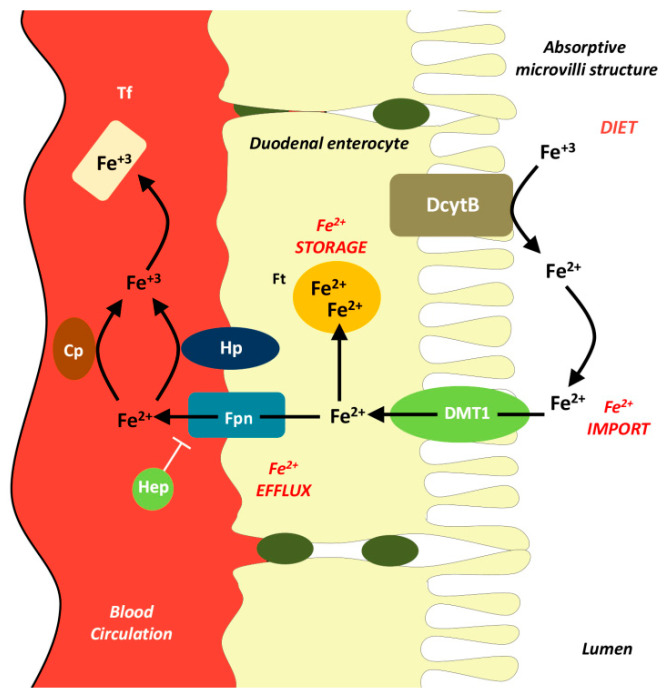
Dietary iron absorption and transport across duodenal enterocytes.

Duodenal enterocytes regulate the levels of circulating Fe through dietary absorption. After absorption in the duodenum and proximal jejunum (Figure 1), iron is transported across enterocytes, reaching the basolateral membrane. Fpn helps irons to cross the basolateral membrane and to enter the systemic circulation. In addition to iron release from enterocytes, Fpn is also responsible for iron export from other cells, including hepatocytes and macrophages [45]. Iron demand also acts as a signal that influences iron absorption from the intestine and/or releases from the reticuloendothelial system since iron deficiency can lead to restricted erythropoiesis and anemia. Increased iron absorption is regulated via enterocytes that synthesize more Fpn, duodenal cytochrome B (Dcytb), and divalent metal transporter 1 (DMT1). Dcytb is needed to reduce ferric Fe^3+^ to Fe^2+^, while DMT1 transports iron into enterocytes, and Fpn releases it into the systemic circulation [46,47,48].

Dietary ferric iron (Fe^3+^) is converted to ferrous iron (Fe^2+^) by the apical ferric reductase duodenal cytochrome b (DcytB). After Fe reduction to the ferrous form (Fe^2+^), it could cross into the cytoplasm by an apical Fe transporting divalent metal transporter-1 (DMT1, also known as DCT1, SLC11a2, and Nramp2). Both DcytB and DMT1 are localized to the microvilli-covered surface of simple columnar and simple cuboidal epithelium, known as the brush border. Iron is either stored or moved from the enterocyte into the circulation through the sole basolateral transporter ferroportin (Fpn, also named SLC40A1). The ferroxidase hephaestin (Hp), aided by ceruloplasmin (Cp), oxidizes Fe^2+^ to Fe^3+^ to enable loading onto the plasma carrier protein transferrin (Tf). Hepcidin (Hep) decreases serum iron levels by inhibiting iron release by Fpn.

Erythroferrone is a Fam132b protein secreted by maturing erythroblasts and functions as a biologically active substance that links erythropoiesis and iron metabolism. There is evidence for the role of erythroferrone as a Hep suppressor in anemias due to blood loss, hemolysis, and hereditary anemias with ineffective erythropoiesis. This ability may be useful for treating anemias with increased hepcidin expression, including anemias in inflammatory diseases, chronic kidney diseases, and iron-deficiency anemias resistant to treatment with iron medications [49]. Erythroferrone deficiency also contributes to the development of insulin resistance in a high-fat diet (HFD), a significant increase in the number of adipocytes, and adipose tissue accumulation, which is associated with an increase in lipoprotein lipase activity [50].

Metabolic syndrome may be associated with elevated transferrin and ferritin levels in about 30% of patients with non-alcoholic fatty liver disease. This phenomenon has been termed “Dysmetabolic Iron Overload Syndrome (DIOS)”. This iron overload can negatively impact metabolic processes and be a risk factor for diabetes mellitus. However, in the process of progression of obesity in such patients, iron deficiency is observed. The development of DIOS is based on an increase in Hep and decreased duodenal Fpn [51].

## 3. Biomarkers of Iron Status

The status and turn-over of Fe are evaluated by numerous characteristics, such as its metabolism, absorption, and interactions with other nutrients. Different methods or biomarkers have been established to determine the supply and status of Fe. Low hemoglobin (anemia) is an ID indicator, although reduced ferritin is considered the best indicator [52]. Serum ferritin is a protein that plays a critical role in Fe storage. The regulation of ferritin synthesis occurs on the post-transcriptional level by binding cytoplasmic iron regulatory protein to an iron-responsive element in the 5′ untranslated region of ferritin mRNA [53]. Ferritin is composed of two subunits: light (L) and heavy (H), having a molecular weight of 19 kDa and 21 kDa, respectively, and homologous sequences. The ratio of L- and H-subunits depends on the tissue type and may impact inflammation or infection. Tissue ferritins might be H-subunit rich, predominately found in the heart and kidney, and L-subunit rich, mostly found in the liver and spleen. Serum ferritin is not the same as tissue ferritin (a heteropolymer of H- and L-subunits). Although serum ferritin is an important clinical marker of iron status, its precise source remains undetermined [54]. Depending upon the Fe content, it contains a molecular weight of ≥440,000 Dalton. It consists of a protein shell (apoferritin) composed of 24 subunits and a Fe core with an average of approximately 2500 Fe^3+^ ions. The ferritin level determination is relevant in diagnosing anemias and in monitoring Fe therapy [55,56]. However, the serum ferritin level is influenced by a series of physiological and pathological factors, such as inflammation, infection, and malignancy [57]. Elevated serum ferritin levels are the key acute-phase reactants and are of great importance to clinicians since they indicate the need for therapeutic interventions to control inflammatory responses in high-risk patients. Although serum ferritin serves as an inflammatory marker, it is unclear whether it reflects or causes inflammation or is involved in an inflammatory cycle. Hyperferritinemia may play a protective role in inflammation because it limits the production of free radicals and mediates immunomodulation [58]. As a key modulator of Fe homeostasis, Hep is considered a promising new biomarker for Fe status, e.g., in chronic kidney disease (CKD) [59]. Reduced values of Hep induce IO in renal disease with ineffective erythropoiesis and also in hereditary hemochromatosis. Due to decreased renal clearance, serum Hep levels increase in CKD. This leads to inhibition of duodenal iron absorption and contributes to systemic iron deficiency, iron deficiency for erythropoiesis, and resistance to endogenous exogenous erythropoietin. As soon as CKD is characterized by impaired renal production of erythropoietin, hepcidin-mediated iron restriction plays a role in CKD patients with anemia [60]. Fnp, Hep and their modulators are proved to be promising targets for diagnosing and treating Fe disorders and anemias [61].

Dysregulation of Hep causes Fe homeostasis modification and the development of pathological disorders, such as Fe restrictive and Fe loading anemias and hemochromatosis [62]. Fe can also be transported in the plasma by transferrin, which donates Fe to cells via the interplay with a specific membrane receptor, named the transferrin receptor (TfR). Although soluble TfR is rarely used clinically, this marker has recently been of interest as a substitute for ferritin in inflammatory processes [63,64,65,66]. Elevated TfR values indicate a depot Fe shortage and functional ID, a condition defined by tissue iron deficiency, despite sufficient iron stores. Combining the biochemical marker ferritin with the soluble TfR, Hep, complemented by other parameters such as transferrin saturation and reticulocyte’s hemoglobin, represents the current measure of repertory in ID and anemia.

## 4. Iron Deficiency and Anemia in Obesity

ID is a common finding of metabolic alterations occurring in obesity [11,67]. A primary underlying pathophysiological mechanism is a decreased ability for duodenal Fe absorption, reported in several studies [21,68,69]. Thus, Mujica-Coopman et al. identified the significantly decreased absorption of isotope-labeled Fe in obese women of childbearing age compared with normal-weight counterparts [70]. The prevalence of IDA among all women studied was approximately 7%, and iron deficiency was observed in 9%. Iron status was normal in 66% of women, with no differences in BMI categories. Although the percentage of Fe absorption was lower in obese women, this did not affect their Fe status. Zimmermann et al. came to a similar conclusion when reporting that a higher body mass index (BMI) is associated with decreased Fe absorption [71]. Among the women included in the study, about 20% had an iron deficiency; this figure among the studied children reached 42%. Iron absorption rates were independent of iron status. Benotti et al. have recently addressed the disturbance in iron metabolism during metabolic surgery in obese subjects [72]. The International Diabetes Federation (IDF) position statement on bariatric surgery has been recommended for treating and preventing T2D in obese people [73,74,75]. The American Diabetes Association talks about “metabolic surgery” to indicate the bariatric approach to prevent and solve T2D in obese subjects [76]. The terminology may be misleading, but the goal is to address a metabolic syndrome with surgery. A meta-analysis by Cheng et al. on iron status in obese populations reported that obese individuals have higher concentrations of ferritin than normal-weight subjects [77], which might result from the low-grade inflammation characterizing obese subjects. The authors of a recent meta-analysis concluded that obese individuals have lower concentrations of serum Fe and decreased transferrin saturation percentages than non-overweight individuals. This meta-analysis also helped to conclude that obese subjects have a considerably higher risk of ID than the controls (OR: 1.31; 95% CI: 1.01–1.68) [22].

The serum Fe reduction phenomenon may be due, in part, to the low-grade or chronic inflammation provoked by the progression of obesity via a chain of pathological mechanisms. Compared to normal-weight subjects, adipose tissue of the obese subjects is characterized by excessive quantities of macrophages and producers of pro-inflammatory cytokines [78]. Moreover, obesity is associated with increased adipokines production in the fat cells that play a central role in regulating insulin resistance and many aspects of inflammation, immunity, and susceptibility to viral infection [79,80]. Dysregulation of adipocytokines production is involved in developing obesity-related diseases, such as diabetes mellitus, hypertension, cardiovascular disease, and hyperlipidemia. Adipocytokines and pro-inflammatory cytokines, together with free fatty acids abundant in obesity, trigger a cascade of harmful adipose tissue reactions to other body systems and organs. Concomitantly, the liver undergoes lipid accumulation (non-alcoholic fatty liver disease, NAFLD), which further disrupts the Fe balance due to an increase in the production of cytokines and insulin resistance [81].

In obese patients, reduced BMI leads to decreased Hep levels, which improves iron absorption and metabolism. After a six-month weight-loss program, these results were observed [82], while inflammatory markers and Fe status were improved after the intervention, which resulted in decreased BMI [83]. Only those weight loss programs based on a well-balanced, healthy approach improved functional Fe status due to increased dietary Fe absorption, decreased expression of inflammatory cytokines, and diminished insulin resistance [21,26,84].

It also has to be pointed out that the treatment of ID might improve obesity status. According to Aktas et al., intake of iron supplements at iron-deficiency anemia significantly reduces BMI, improves waist circumference, and decreases triglyceride after treatment compared to the pre-treatment period [85]. On the contrary, iron deficiency anemia aggravates obesity since it is associated with a greater state of fatigue, which results in a further decrease in physical activity [86]. There is no doubt that ID must be identified and adequately controlled in all individuals suffering from overweight and obesity.

## 5. Iron Deficiency, Inflammation, and Obesity

There is a close relationship between Fe status change and inflammatory activity. For example, it has been shown that pro-inflammatory cytokines such as interleukin-6 (IL-6) can increase the contents of Hep in liver cells. IL-6 stimulates Hepcidin Antimicrobial Peptide (HAMP) expression, and this effect is mediated via a signal transducer and a transcription activator binding site on the Hep promoter [87]. Increased inflammatory activity with reduced intestinal absorption of Fe, with stronger sequestration of Fe in macrophages and lowered Fe, are reflected in the serum, which, at the same time, shows an increase in serum ferritin levels [88,89,90].

The subsequent decrease in hemoglobin and Fe levels of plasma induces the anemia referred to as anemia of inflammation. These Fe metabolism modifications likely play a critical role in host defense by restricting the Fe availability for invading microorganisms [91]. This relationship between inflammatory activity and ID is known to be of global importance because of the prevalence and incidence of some situations, such as overweight, obesity, and conditions with chronically elevated inflammatory activity [24]. In the case of overweight, increased levels of circulating IL-6 are also found [92].

Chronic inflammation is considered one of the processes linked to obesity and obesity-associated diseases, including insulin resistance [93]. Obesity is accompanied by inflammation of body fat, which proceeds with adipose tissue infiltration by immune-competent cells. This process is associated with hypersecretion of TNFα and IL-6 and the development of insulin resistance, activated inflammation of colon macrophages, and their recruitment into adipose tissue [94].

One recently launched point of view states that several activators of the immune system, including smoking, a surplus of saturated trans-fats, omega-6 fatty acids, and carbohydrates with a high glycemic index, together with a sedentary lifestyle, promote the development of obesity. The result may be a cascade leading to insulin resistance and atherosclerosis [95,96,97]. Acute-phase proteins, including coagulation proteins (fibrinogen, prothrombin) and transport proteins (including ceruloplasmin, haptoglobin, ferritin, and C-reactive protein), act as mediators of the immune response. Interleukins increase the synthesis of acute-phase proteins and components of the complement system in the liver, and their elevated level in the serum is a sign of systemic inflammatory response [92].

Triggering factors for the synthesis of acute-phase reactants are adipokines, among which the most studied are tumor necrosis factor-alpha (TNFα) and IL-6. TNFα is synthesized not only by macrophages but also by adipocytes and stromal cells. Its concentration in tissues is hundreds of times higher than in blood; the local effects include stimulation of lipogenesis and adipocyte growth. TNFα has systemic effects by activating fatty acids’ synthesis and increasing their concentration in the blood [98,99,100].

About 30% of circulating IL-6 is synthesized by fat cells. The study included 22 women and 17 men: median age, 36 years (interquartile range, 26–48 years); body mass index, 31.8 kg/m^2^ (range, 22.3–38.7 kg/m^2^); percent body fat, 28.7% (range, 17.6–50.7%). IL-6 has been released from the adipose tissue bed of all subjects. However arterial plasma concentrations of IL-6 were correlated significantly with body mass index (Spearman’s r = 0.48; *p* < 0.01) and percentage of body fat (Spearman’s r = 0.49; *p* < 0.01) [101].

A positive relationship between various anthropometric parameters of obesity and plasma levels of IL-6 has been described for men and postmenopausal women (estrogens are known to inhibit IL-6 secretion) [102,103,104].

In individuals with severe obesity, elevated blood leptin levels and leptin resistance are usually observed [105]. Recent studies have shown that increased leptin levels contribute to oxidative stress by enhancing macrophages’ phagocytic activity and inducing the synthesis of pro-inflammatory cytokines (TNFα, IL-6, IL-2, and interferon-gamma) and secondarily increased level of endothelial cell dysfunction markers [106,107].

Another adipokine, visfatin, is synthesized by bone marrow and blood lymphocytes and is also present in fat tissue [108,109]. Visfatin has prooxidant and pro-inflammatory effects [110]. The inflammation in obese subjects is closely related to ID, and it induces impaired Fe absorption in the duodenum with inhibition of duodenal ferroportin expression and increased Hep concentrations [21]. In obese people, biomarkers such as ferritin, soluble TfR, and Hep are more susceptible to oxidative damage, directly related to BMI, fat percentage, and triglyceride levels [111]. Antioxidant protection markers are significantly reduced, along with the development of obesity, particularly in the central type, characterized by significant fat deposition in the abdomen [112].

## 6. Bariatric Surgery and Postoperative Iron Status

Bariatric surgery is the most effective treatment for weight loss and long-term weight maintenance. It improves life quality by reducing obesity-related comorbid conditions such as cardio- and cerebrovascular diseases, respiratory diseases, T2D, degenerative joint disease, and even cancer [113]. The bariatric procedures comprise gastric banding, sleeve gastrectomy (SG), Roux-en-Y gastric bypass (RYGB), and biliopancreatic diversion (BPD), with or without duodenal switch (DS). Of these methods, SG is the most common surgical weight-loss procedure. As discussed above, ID and anemia are frequent in obese patients.

Consequently, monitoring the Fe status before bariatric surgery is of crucial importance [28,114]. Bariatric patients with anemia generally stay longer in hospital than nonanemic patients (2.7 vs. 1.9) [115]. Iron loss following bariatric surgery is also expected to enhance absorption unless absorptive capacity is concomitantly reduced, resulting from bariatric surgery. Thus, after bariatric surgery, anemia often becomes a significant concern [114,115]. Some bariatric surgery methods, such as biliopancreatic diversion, duodenal switch, and RYGB, have been associated with a malabsorption procedure, which leads to impaired absorption of Fe. Patients after surgery are characterized by a decrease in the transition of Fe^3+^ to Fe^2+^ due to hydrochloric acid deficiency leading to anemia [116]. Together with decreased secretion of hydrochloric acid, different factors of anemia and ID are attributed to the reduced food intake and frequent occurrence of meat intolerance [28,117,118]. The American Society for Metabolic and Bariatric Surgery (ASMBS) recommended guidelines to recover iron levels following bariatric surgery [28,119,120]. A study in a group of 32 women who underwent bariatric surgery and post-bariatric abdominoplasty showed that, two days after surgery, the average hemoglobin level decreased from 12.98 to 10.8 g/dL. Seven days later, it increased to 11.53 g/dL, but there was no further increase in hemoglobin. The same trend is found for serum Fe and transferrin. The average ferritin level decreased within 56 days after surgery from 29.8 to 16.4 μg/L. Iron and hemoglobin deficiency were observed in 45% of the patients [121].

Moreover, ID anemia may increase over time in patients after bariatric surgery, even if they take Fe supplements. A ten-year follow-up of a group of 151 patients after gastric bypass surgery, conducted in Brazil, showed that anemia persisted in 37.5% of the patients when the ferritin level was lower than 15 μg/L, and in 45.0% when the ferritin was lower than 30 μg/L [122]. In a Portuguese retrospective cohort study involving 1999 patients with a follow-up period of 4 years, post-bariatric surgery anemia was diagnosed in 24.4% of the patients. The variables associated with a higher prevalence of anemia were sex and the type of bariatric surgery. Females and RYGB procedures present a two-fold increased risk of developing anemia compared to males and gastric sleeve and gastric band surgery [123]. Of the studied patients, 84.8% were female, with a median age of 42.3 years. These findings are consistent with the results of another study that showed similar results: the risk of developing anemia was three-fold higher in women than men [124].

On the other hand, given that obese patients have elevated serum Hep levels and signs of inflammation associated with obesity, it can be expected that a decrease in the amount of adipose tissue after bariatric surgery can activate Fe absorption [125]. This assumption was demonstrated by the results of a six-month prospective study in 38 patients who underwent laparoscopic sleeve gastrectomy (LSG). Patients consumed iron sulfate (6 mg ^57^Fe) and intravenous iron citrate (100 μg ^58^Fe). Six months later, a decrease in body fat, interleukin IL-6, and Hep was found (*p* < 0.005 for all indicators). Iron absorption increased by 30% in patients with ID (from 9.7% to 12.4%, *p* = 0.03), while, in individuals with normal Fe content, absorption remained unchanged. The results allowed the authors to conclude that loss of adipose tissue leads to improved absorption of Fe [126]. However, gastric bypass surgery, especially RYGB and sleeve gastrectomy, induces iron malabsorption that could accentuate ID [127,128].

## 7. Iron Supplementation after Bariatric Surgery

In general, in symptoms including performance weakness from fatigue, irritability, and apathy, the ID diagnosis should always be considered, even if there is no anemia. Increasing public awareness exists to improve nutrition and use food supplements when needed, such as Fe supplements [12]. Simultaneous consumption of vitamins and minerals, abundant in fruits or fruit juices, improves the Fe absorption. If these measures are not sufficient, ID can be cured through food supplements, medication, and intravenous drug therapy [129,130]. Given new findings concerning Fe metabolism regulation and the analytical availability of the corresponding biomarkers (Ft, soluble TfR, Hep), it is possible to detect an insufficient supply or a disturbed balance in Fe metabolism at an early stage. This is particularly important among women of reproductive age to optimize the maternal nutritional status before pregnancy and during the prenatal course [131]. Drug therapy can be performed orally or, in some cases, parenterally. If possible, iron should be administered orally, and intravenous administration should be considered when oral iron is insufficient or not well tolerated [132]. However, there is high variability in iron supplementation strategies among clinicians. In the systematic review of Enani et al., the iron supplementation dosage varied from 7 to 80 mg daily across the studies evaluated [127]. After bariatric surgery at Innlandet Hospital, Norway, patients are routinely recommended daily supplementation with Fe (100 mg), usually in the form of sulfate. In a cross-sectional study of 36 women with an average age of 45 years who underwent bariatric surgery, ID was found in 42% of participants. The additional administration of non-heme Fe at a dose of 45 mg/day had a positive association with serum ferritin (β = 0.964; *p* = 0.029). Most recent studies demonstrate that bariatric surgery effectively normalizes menstrual regularity in 74–85% of obese women of reproductive age, correlated with weight loss [133,134,135]. The intake of vitamin C from food also contributed to an increase in Fe levels [136]. The Obesity Society and American Society for Metabolic and Bariatric Surgery recommend a dose of 195 mg non-heme iron (sulfate, fumarate, or gluconate) per day for bariatric surgery patients [137]. A recent RCT (NCT 02404012) indicated that this dose of non-heme iron is effective for normalization of Fe status following RYGB, whereas a commercial heme iron supplementation (in a polypeptide form and in a recommended dose of 31.5 mg/day) proved ineffective in this regard [138]. Interestingly the bioavailability of such an oral formulation was greater in healthy subjects compared to ferrous sulfate. However, even in patients with chronic kidney disease, its efficacy was not superior [138].

Further studies are needed to find new heme iron formulations with greater water solubility and efficacy in improving iron status biomarkers, even in RYGB patients. Ferrous sulfate is the gold standard in oral iron supplements for treating ID in bariatric surgery patients, but it is not always well-tolerated. Gastrointestinal complaints and nausea are not uncommon. These can be mitigated by using fortified foods. For patients with severe oral iron intolerance or severe ID due to iron malabsorption, intravenous iron infusion (dextran, ferric gluconate, or sucrose) is necessary [137]. A recommended form of Fe is a sustained-release preparation. A slow-release formulation based on Fe-Kojic acid complexes is better absorbed in patients with sleeve gastrectomy but not in those with gastric bypass [139]. Chewable supplements with multivitamins and minerals are available. They should contain at least 18 mg of iron. Vitamin B_12_ and fat-soluble vitamins A, D, E, and K are included, together with microelements such as selenium, copper, and zinc. Calcium decreases iron absorption and should be taken separately (as citrate [140]) two hours apart from iron. The dose to be taken (1, 2, or 3 tablets/day) is related to the type of bariatric surgery (SG, RYGB, or DS, respectively). Close monitoring and tailored Fe supplementation pre-and post-bariatric surgery is required, and lifelong monitoring performed under appropriate laboratory supervision is recommended [141,142].

## 8. Concluding Remarks and Future Perspectives

Iron deficiency is the most crucial micronutrient deficiency known in children, and it has received growing attention as a global public health issue. Such a deficiency is commonly related to low Fe intake or increased physiological demands, as seen in pregnancy, chronic inflammatory diseases, including morbid obesity, or after bariatric surgery. Different studies have shown that the determination of several biomarkers is necessary to evaluate individual Fe status. The current data reveal multiple forms of interactions between Fe and the immune system. These interactions may critically impact the Fe status in ID, which is frequently observed in obese individuals. This review has summarized the role of pro-inflammatory cytokines, such as interleukin-6, which can increase Hep synthesis in liver cells and inhibit Fe absorption.

Moreover, we explored the burden and characteristics of ID and anemia in obese patients and after bariatric surgery. In cases of morbid obesity remitted for bariatric surgery, it is mandatory to evaluate Fe status, both pre- and postoperatively, with long-life proper monitoring in an appropriate clinical context. Furthermore, monitoring the effect of supplementation is also needed to avoid Fe excess. Aside from the fundamental role of Fe in anemia, a less focused issue regards the role of Fe as a micronutrient in many other biological activities of the organism. The present report summarizes the outcomes of different analyses, which reveal that Fe deficiency remains a primary global health objective.

## Abbreviations

BMI: body mass index, BPD: biliopancreatic diversion, DMT1: divalent metal transporter-1, DS: duodenal switch, Fpn-1: ferroportin-1, Ft: ferritin, Hep: hepcidin, Hp: hephaestin, ID: iron deficiency, IL-n: interleukin-n, IO: iron overload, LSG: laparoscopic sleeve gastrectomy, RYGB: Roux-en-Y gastric bypass, SG: sleeve gastrectomy, T2D: Type 2 Diabetes, Tf: transferrin, TNFα: tumor necrosis factor-alpha.
